# Supplementary material for: Prevalence of uremic neuropathy and the effect of dialysis in children with end-stage renal disease: A cohort study
Source: PLoS One. 2025 Nov 25;20(11):e0337696. doi: 10.1371/journal.pone.0337696 (PMC12646417; doi:10.1371/journal.pone.0337696)
Supplement: Attachment — Submitted filename: Response to Reviewers 2.pdf [file pone.0337696.s003.pdf]

Response to Reviewers

Manuscript ID: PONE-D-25-17216R1
Title: *Prevalence of uremic neuropathy and the effect of dialysis in children with end-stage renal disease: a cohort study*

Dear Academic Editor and Reviewer,

We sincerely thank you for your valuable time and constructive comments that helped us improve the quality and clarity of our manuscript. Below we address each comment point-by-point. All modifications have been highlighted in the revised manuscript.

Journal requirement:

The reference list was reviewed and updated to ensure accuracy and currency; no cited articles have been retracted.

Reviewer #3

Comment 1: Authors indicate that the study aimed to assess UN in children ESRD in a North African population. The study was conducted in Tunisia, as far as I understand. Therefore, it would be better if the reference to North African population is removed everywhere and replaced by Tunisia as one of the North African countries.
Response: We thank the reviewer for this helpful clarification. We have replaced all mentions of "North African population" with "Tunisian population" throughout the manuscript.

Comment 2: Line 80 – please change to "Study Design."
Response: Corrected as suggested.

Comment 3: In the Study Design section, please indicate where the study was conducted.
Response: We have added the location: "followed in Department of Pediatric Nephrology at University Hospital of Sahloul (Tunisia)."

Comment 4: Line 85 – please define Stage 5 chronic kidney disease, what are the criteria?
Response: We had already defined Stage 5 CKD in the Introduction (line 63-64) as "an eGFR < 15 mL/min/1.73 m² according to KDIGO 2024 criteria." To improve clarity, we have now also repeated this definition briefly in the Study Design section (line 85) and updated the terminology to align with the KDIGO classification system (CKD G5, CKD G3..).

Comment 5: Lines 107–110 – please define what T1 and T2 mean.
Response: We added the clarification: "T1 refers to baseline assessment; T2 refers to the 6-month follow-up evaluation."

Comment 6: In the ethics section, indicate whether assent from minors was obtained.
Response: Children were free to participate, decline, or withdraw from the EMG study at any

time. We respected the child's wishes, and any examination was immediately stopped if the child showed signs of discomfort or refusal. The EMG protocol was limited to a few nerves, and stimulation intensity was kept below 25 mA for motor nerves and 15 mA for sensory nerves to avoid activation of nociceptive fibers.

Comment 7: Line 180 – please adjust SD formatting.
Response: We corrected all SD expressions as suggested (now "-0.84 (SD ±1.2; range: -2.7 to 2.4)").

Comment 8: For weight-for-age, height-for-age, BMI-for-age, indicate normal values.
Response: We have added reference ranges according to WHO growth standards (2007).

Comment 9: Please define the abbreviation "PTH."
Response: Defined as "parathyroid hormone (PTH)" at first mention.

We appreciate the reviewer's positive evaluation and believe the revised version addresses all minor comments.

Sincerely,
Dr. Arwa Yahyaoui, on behalf of all co-authors
