# Supplementary material for: Prevalence of uremic neuropathy and the effect of dialysis in children with end-stage renal disease: A cohort study
Source: PLoS One. 2025 Nov 25;20(11):e0337696. doi: 10.1371/journal.pone.0337696 (PMC12646417; doi:10.1371/journal.pone.0337696)
Supplement: Attachment — Submitted filename: reviewers response .docx [file pone.0337696.s002.docx]

## Response to Reviewers

Dear Editor,

Thank you very much for revising our manuscript (ID: PONE-D-25-17216)

We have carefully revised the manuscript in response to the comments we received. We are pleased that there is interest in the topic and are grateful for the constructive comments.

We have addressed the comments point by point and have made corresponding changes in the manuscript in track changes.

Appended below, please find the list of all reviewers' comments and our response to each point.

We hope this revised version will be suitable for publication in the journal.

Yours sincerely,

Corresponding author: Dr. Arwa Yahyaoui

arwayahyaoui.ay@gmail.com

- **Journal Requirements :**

1. **Please ensure that your manuscript meets PLOS ONE's style requirements, including those for file naming.**

I rectified the manuscript as well as the title page according to the journal requirements.

2. **We note that your Data Availability Statement is currently as follows: All relevant data are within the manuscript and its Supporting Information files.**

Data are available upon request from the corresponding author

### ****Response to Reviewer #1:****

**General Comment:**
This is a good article The topic of the article is also interesting and new. The topic of neuropathy in children and kidney diseases is very important and can have a great impact on the prevention and treatment of the disease. Good luck.

**Response:**
We sincerely thank Reviewer #1 for their positive feedback and encouragement. We are pleased that the relevance and novelty of our work on pediatric neuropathy in kidney disease were recognized. Your supportive comments are appreciated.

### ****Response to Reviewer #2:****

### 1. ****“The introduction and title are weakly consistent.”****

We fully understand this concern. The introduction has been rewritten to better define the clinical context of uremic neuropathy in children and to clearly articulate the study’s objectives. We aimed to make the rationale more explicit and to show how our work fills a gap in current pediatric nephrology research.

In line with this, we revised the title to better reflect the scope and key outcomes of our study. The new title is “***Prevalence of uremic neuropathy and the effect of dialysis in children with end-stage renal disease: a cohort study”***

### 2. “The type of study was not well chosen.”

We appreciate this comment and would like to clarify our choice. The objective of our study was to assess both the prevalence of peripheral neuropathy and its progression in relation to dialysis. To achieve this, we used a **prospective cohort study**, conducted from July 2021 to September 2023. All included patients with ESRD stage V were followed over time. At baseline, all underwent nerve conduction studies (EMG), and were then monitored for 6 months with repeat EMG to evaluate changes in neuropathy status

Patients were divided into two predefined exposure groups at inclusion:

- Dialysis group: children undergoing renal replacement therapy (hemodialysis)
- Pre-dialysis group: children managed conservatively

The prospective design allowed us to compare the evolution of uremic neuropathy between groups, testing the hypothesis that dialysis is associated with improvement in neuropathy over time. This approach is appropriate for studying risk factors and treatment effects in rare pediatric populations.

We have now made this rationale clearer in the revised **Methods** section.

### 3. ****“The methodology has many shortcomings and is not acceptable.”****

We acknowledge that the original methodology section lacked some important details. In the revised version, we have addressed the following points:

- We clearly defined our inclusion and non-inclusion criteria;
- We have now added a **sampling section** to the Methods to clarify our approach
- We provided a detailed description of the nerve conduction study protocol, referencing accepted pediatric guidelines;
- We described how neuropathy was diagnosed clinically and electrophysiologically;
- We explained how the severity of abnormalities was graded and how follow-up EMG changes were evaluated.

We believe these clarifications improve the scientific rigor of our study and make it easier for readers to assess the validity of our findings.

### 4. ****“The sample size was incorrectly determined and is too small.”****

Thank you for highlighting this issue. The sample size was calculated to estimate the prevalence of peripheral neuropathy in children with ESRD with adequate precision. We assumed a prevalence (p) of 22%, based on published pediatric data [7]. We chose a confidence level of 95% (Z = 1.96) and an absolute precision (d) of 5%.

The initial sample size for an infinite population was calculated using the standard formula for proportions: n=d2Z2×p×(1−p)

Given that the total eligible population of children with ESRD in Tunisia was estimated at N = 170 [11], we applied the finite population correction

However, due to strict exclusion criteria (excluding children with neuropathy from other causes) and limited recruitment feasibility, the final enrolled cohort consisted of 31 children over 2 years. While this number is lower than the adjusted target, it reflects the practical constraints of a rare disease setting, the high burden of comorbidities excluding eligibility, and ethical considerations in pediatric research.

Importantly, our study retains value by reporting prevalence estimates with appropriate confidence intervals and serving as a first prospective study of this condition in this population.

We have now included a detailed explanation of this calculation in the **Methods** section and addressed the implications of the small sample size in the **Discussion**

### 5. ****“Due to the small sample size, most statistical analyses and results are incorrect.”****

We respectfully disagree with this conclusion but understand the concern. The statistical analysis was carefully conducted using methods appropriate for small sample sizes. We tested for normality, applied non-parametric tests when necessary, and used exact p-values.

That said, we have now more clearly described the tests used, their justification, and the assumptions checked. We have also acknowledged in the **Discussion** that the small sample limits statistical power and that our findings should be interpreted with caution.

### 6. ****“The discussion is very poorly written.”****

We thank the reviewer for this honest feedback. The **Discussion** section has been completely rewritten. It now follows a clearer and more logical structure:

- Summary of key findings;
- Comparison with relevant literature;
- Interpretation of results;
- Clinical implications;
- Limitations and suggestions for future research.

We made an effort to avoid repetition and ensure each paragraph adds meaningful interpretation to the results.

### Additional Revisions:

- Language and grammar have been revised throughout the manuscript for clarity and fluency;
- References were updated and expanded to include recent and relevant studies;
- The tone was adjusted to be more objective and aligned with academic standards.

We hope the revised manuscript now meets the expectations of the reviewers and the editorial board. We remain available for any further clarification you may require and sincerely thank you again for your constructive and thoughtful feedback.

Warm regards,
**Dr. Arwa Yahyaoui**
On behalf of all co-authors
